# Efficacy and safety of Chinese herbal footbaths for the treatment of dysmenorrhea: Protocol for a systematic review and meta-analysis

**DOI:** 10.1371/journal.pone.0250685

**Published:** 2021-05-03

**Authors:** Min Xiao, Lizhou Liu, Steve Tumilty, Dan Liu, Yanyan You, Yunhui Chen, Songqi Tang, Wei Huang, George David Baxter

**Affiliations:** 1 College of Clinical Medicine/College of Basic Medicine, Chengdu University of Traditional Chinese Medicine, Chengdu, Sichuan, China; 2 Centre for Health, Activity, and Rehabilitation Research, School of Physiotherapy, University of Otago, Dunedin, Otago, New Zealand; 3 West China Hospital, Sichuan University, South Renmin Road, Wu Hou District, Chengdu, Sichuan, China; 4 College of Traditional Chinese Medicine, Hainan Medical University, Haikou, Hainan, China; Universita degli Studi di Napoli Federico II, ITALY

## Abstract

**Background:**

Chinese herbal footbaths are an external therapy of traditional Chinese medicine that has been widely used to treat dysmenorrhea. This review aims to systematically evaluate its efficacy and safety for the treatment of dysmenorrhea.

**Methods:**

Databases of PubMed, EMBASE, Cochrane Library, CIHAHL, Web of Science, Chinese National Knowledge Infrastructure(CNKI), Chinese Scientific Journals Database (VIP), Wanfang Database, China Biomedical Literature Database(CBM), and Chinese Biomedical Literature Service System (SinoMed) will be searched from the inception to September 30, 2020. The eligible randomized controlled trials (RCTs) will be identified and included. The primary outcomes include pain intensity measured by validated scales of visual analog scale, numeric rating scale, and response rate of symptom reduction. The secondary outcomes are scores on validated pain questionnaires, quality of life measured by SF-36 or other validated scales, and adverse events. Study selection, data extraction, and assessment of bias risk will be conducted by two reviewers independently. RevMan software (V.5.3.5) will be utilized to perform data synthesis. Subgroup and sensitivity analysis will be performed when necessary. The strength of the evidence will be evaluated with the Grading of Recommendations Assessment, Development and Evaluation System.

**Results:**

A high-quality synthesis of current evidence of Chinese herbal footbaths for patients with dysmenorrhea will be provided in this study.

**Conclusion:**

This systematic review will provide evidence of whether Chinese herbal footbaths are an effective and safe intervention for the treatment of dysmenorrhea.

**Systematic review registration:**

PROSPERO CRD42020188256.

## Background

Dysmenorrhea refers to the throbbing or cramping pain in the lower abdomen occurring just before and during menstruation [1]. It is one of the most common gynecologic disorders and classified as primary or secondary dysmenorrhea based on pathophysiology. Primary dysmenorrhea is defined as menstrual pain without pelvic pathology, and secondary dysmenorrhea is usually due to identifiable organic pathologies, such as adenomyosis, endometriosis, and pelvic inflammatory diseases [2]. The prevalence of dysmenorrhea varies between 16% and 91% among women of childbearing age, while the prevalence reaches 80% in adolescents [3, 4]. It is frequently accompanied by nausea, bloating, vomiting, fatigue, diarrhea, headache, lower backache, and dizziness [5]. This ailment negatively influences women’s daily activities, impairs sleep quality, associates with anxiety and depression, represents a risk factor for fibromyalgia, lowers academic performance in adolescents, and decreases effectiveness in women’s daily tasks [6–9]. In severe cases, approximately 3 to 33% of women are absent from work or school for 1 to 3 days each cycle [10, 11]. In the United States alone, dysmenorrhea causes the loss of 600 million working hours and two billion dollars per year [12]. Hence, dysmenorrhea has been considered a public health matter with socio-economic impacts and calls for an increased need for medical care [13].

Previous studies indicate that the overproduced prostaglandins (PGs) may contribute to the pathomechanisms of dysmenorrhea. PGs are synthesized from arachidonic acid through the cyclooxygenase (COX) pathway. Increased PGs cause hypercontractility and hypoxia of uterine smooth muscle with decreased uterine blood flow and elevated sensitivity of peripheral nerves to pain [14]. Multiple factors such as vasopressin, oxytocin, calcium, oxidative stress, inflammation, and nitric oxide have also been implicated in the mechanism [15–17]. Currently, non-steroidal anti-inflammatory drugs (NSAIDs) are considered the first-line treatment for dysmenorrhea; however, they are ineffective for or intolerable by approximately 15% of patients and may cause gastrointestinal and neurological adverse effects and a higher risk of severe cardiovascular disease with long-term usage [18, 19]. Other options including oral, intravaginal, and intrauterine hormonal contraceptives are generally applicable only to the patients with the desire of contraception [20]. Hence, in recent years, there has been growing interest in integrative medicine, and more and more patients turn to traditional Chinese medicine (TCM) for help [21, 22]. Chinese herbal footbaths are an external therapy of TCM and have been developed and utilized in China to treat various diseases for over 3,000 years [23].

In the therapy of Chinese herbal footbaths, patients with dysmenorrhea are required to soak the feet and legs in a hot herbal infusion for 20 to 30 minutes. It is not just for relaxation, but more importantly, a comprehensive approach that integrates reflection effects, thermal effects, and pharmacological actions of Chinese herbs. In the theoretical system of TCM, the feet have channels and acupuncture points that correspond to viscera and different parts of the body, and Chinese herbs absorbed through the skin and mucosa can act on the channels, acupuncture points, and viscera to relieve pain [24]. Chinese herbal formulas such as *Shaofu Zhuyu Tang* (Foeniculi Fructus, Zingiberis Rhizoma, Corydalis Rhizoma, Myrrha, Rhizoma Chuanxiong, Angelicae Sinensis Radix, Radix Paeoniae Rubra, Cortex Cinnamomi, Typhae Pollen, and Trogopteri Feces), *Siwu Tang* (Radix Rehmanniae Praeparata, Radix Angelicae Sinensis, Rhizoma Ligustici Chuanxiong, and Radix Paeoniae Alba), and *Danggui Shaoyao San* (Paeoniae Radix Alba, Angelica Sinensis Radix, Atractylodis Macrocephalae Rhizoma, Chuanxiong Rhizoma, Alismatis Rhizoma, and Poria) are commonly used to treat dysmenorrhea effectively based on pattern differentiation. Their pharmacologic effects on analgesia, spasmolysis, microcirculation, anti-inflammation, vasodilatation, and neuroprotection have been well-documented. Experiments have shown these herbal formulas can modulate PGE_2_ and PGE_2α_ production, block calcium channel and COX-2, increase the expression of nitric oxide and its synthetase, downregulate the contents of oxytocin, vasopressin, endothelin-1, and malondialdehyde, reverse the increased superoxide, and decrease the levels of interleukin-6, tumor necrosis factor-2α, whole blood viscosity, and plasma viscosity [25–30]. Further, footbaths can also improve microcirculation and increase skin permeability to enhance the absorption of TCM herbal formulas and their active compounds [31, 32].

In recent years, a growing body of clinical trials has been conducted to assess the outcomes of Chinese herbal footbaths for the treatment of dysmenorrhea, and the results have suggested it might be an effective and safe therapeutic approach. However, currently no systematic review and meta-analysis have been reported for this specific malady. In this study, eligible randomized clinical trials (RCTs) will be included and systematically synthesized without language and publication restrictions. To the best of our knowledge, this meta-analysis is the first attempt to assess the available evidence of Chinese herbal footbaths for the treatment of dysmenorrhea. Hopefully, the findings of this study may provide helpful evidence for the patients, physicians, and investigators concerned.

## Methods

### Study registration

This systematic review protocol has been registered on PROSPERO (www.crd.york.ac.uk/prospero/) with number PROSPERO CRD42020188256. It will be performed in accordance with the reporting guidelines and criteria set in the Preferred Reporting Items for Systematic Reviews and Meta-analyses (PRISMA) statement checklist (S1 Checklist) [33].

### Eligibility criteria

#### Types of studies

Randomized controlled trials (RCTs) that evaluated the effectiveness and safety of Chinese herbal footbaths for dysmenorrhea will be included. No restrictions will be made on publication status, language, or year of publication. Non-RCTs, reviews, animal-based research, conference proceedings, and literature review will be excluded.

#### Participants

Patients diagnosed with dysmenorrhea using any recognized diagnostic criteria will be included regardless of age, the source of cases, and the duration and severity of the disease.

#### Types of interventions

Dysmenorrhea patients are treated with Chinese herbal footbaths, either alone or in any combination with conventional treatments and other administrative forms of Chinese herbal medicine, will be included. There is no restriction regarding the conventional regimen.

#### Types of comparisons

Dysmenorrhea patients treated with conventional (the same conventional regimen as intervention group in the same original study), a different administrative form of herbal medicine (such as decoction and pills), placebo, or no treatment will be included.

#### Types of outcomes

The primary outcomes include pain intensity measured by validated scales of visual analog scale [34], numeric rating scale [35], and response rate of symptom reduction. The secondary outcomes are scores on validated pain questionnaires [36], quality of life measured by SF-36 or other validated scales [37], and adverse events.

### Information source and search strategy

Ten databases including the PubMed, EMBASE, Cochrane Library, CIHAHL, Web of Science, Chinese National Knowledge Infrastructure(CNKI), Chinese Scientific Journals Database (VIP), Wanfang database, China Biomedical Literature Database(CBM), and Chinese Biomedical Literature Service System (SinoMed) will be searched from the inception to September 30, 2020. Two reviewers will search the literature independently. The following terms will be used in a combination for the electronic search: dysmenorrhea, menstrual pain, painful menstruation, period pain, painful period, menstrual cramps, menstrual disorder, pelvic pain, menstrual cramps, painful menstrual periods, Chinese herbal footbaths, bath, hydrotherapy, herbal bathing, lavipeditum, randomized controlled trial, randomized, randomly, trials, and RCT. Any inconsistency will be solved by a third reviewer. Manual searches will be performed for relevant studies found in the reference lists of included studies. The search strategy for PubMed is presented in Table 1 and will be modified upon the requirement of other databases.

**Table 1:** Search strategy for the PubMed.

No.	Search terms
#1	dysmenorrhea
#2	menstrual pain
#3	painful menstruation
#4	period pain
#5	painful period
#6	cramps
#7	menstrual disorder
#8	pelvic pain
#9	menstrual cramps
#10	painful menstrual periods
#11	#1 OR #2 OR #3 OR #4 OR #5 OR #6 OR #7 OR #8 OR #9 OR #10
#12	Chinese herbal footbaths
#13	bath*
#14	hydrotherapy
#15	herbal bathing
#16	lavipeditum
#17	#12 OR #13 OR #14 OR #15 OR #16
#18	#11 AND #17
#19	randomized controlled trial
#20	randomized*
#21	randomly*
#22	trials
#23	RCT
#24	#19 OR #20 OR #21 OR #22 OR #23
#25	#11 AND #18 AND #24

*Represent one or more characters of all characters.

### Data collection and analysis

#### Study selection

**Fig 1:**
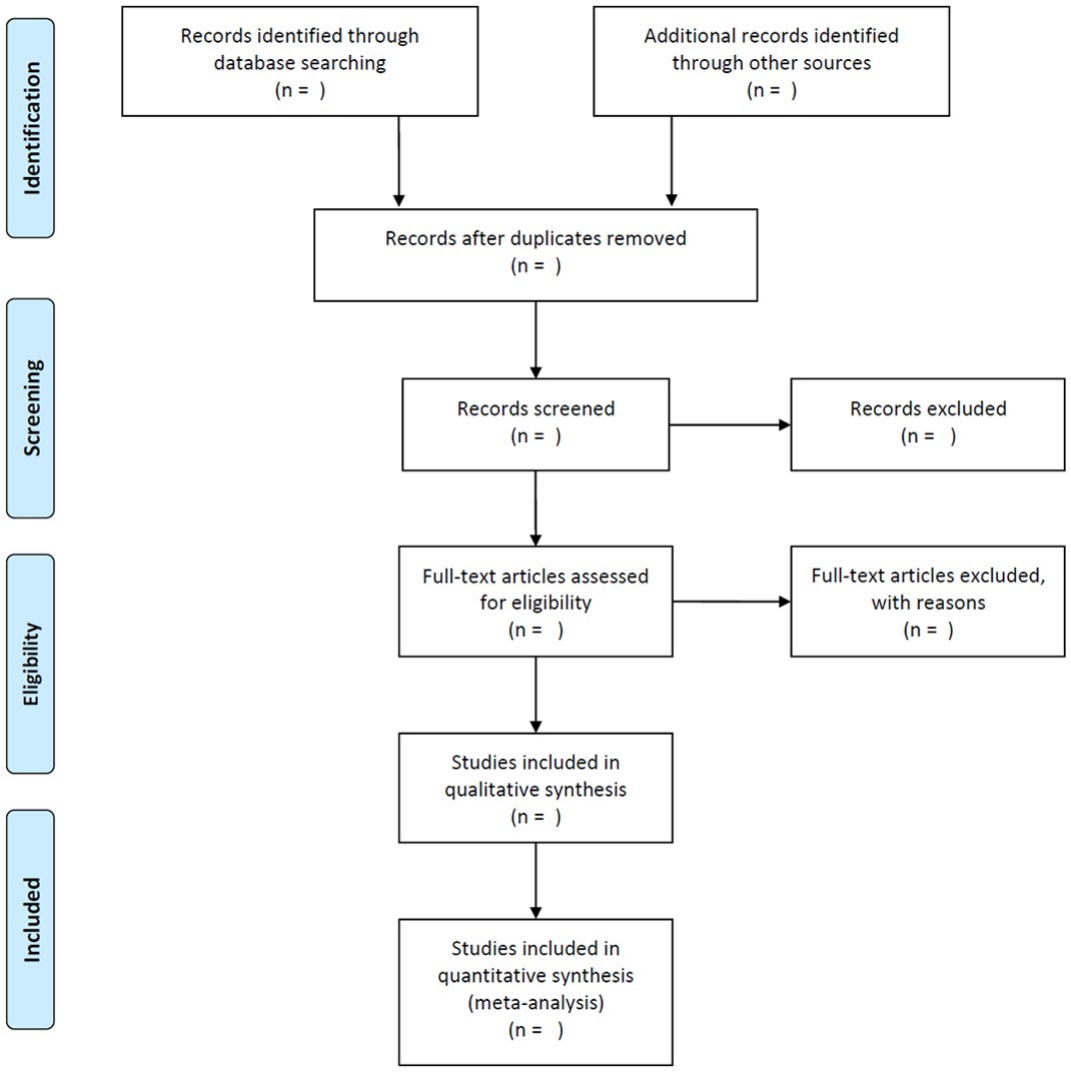
Flowchart of study selection.

Tasks of screening, study selection, and data extraction will be performed independently by two reviewers. The literature will be input into the EndnoteX9 to screen the title and abstract, and the duplications and ineligible studies will be excluded. The final eligible studies will be included after reading the full text of the remaining studies. The corresponding author will be contacted if the full text is not available. Disagreements will be resolved by a third reviewer. The entire process of study selection is presented in a PRISMA flow chart (Fig 1).

#### Data extraction and management

The data extraction will be conducted by two reviews independently in a standard excel spreadsheet, including characteristics of reference ID, author information, year of publication, study type, study design, setting of study, sample size, participant characteristics (age, duration and severity of dysmenorrhea, etc.), Chinese herbal footbaths intervention group and control group (details of Chinese herbal footbaths formulation, randomization, blinding, allocation, intervention approach and duration), and primary and secondary outcome measurements. Studies with less than 30 participants will also be included. Disagreements will be solved by discussion. If the data provided in the research is unclear, missing, or difficult to be extracted reliably, the corresponding author will be contacted by email for clarification. All data will be cross-checked and transferred to RevMan software (V.5.3).

#### Assessment of risk of bias

Two reviewers will independently use the Cochrane risk-of-bias assessment tool to grade the risks of bias as high, unclear, or low risk of bias in terms of the following domains: randomization sequence generation, randomization allocation concealment, blinding of participants, blinding of personnel, blinding of outcome assessors, incomplete outcome data, selective reporting, and other bias. A third reviewer will be consulted for inconsistency. Funnel plots will be conducted to assess publication bias when the included studies are more than 10. If feasible, Egger regression and Begg correlation test will be conducted to identify the funnel plot asymmetry.

#### Measures of treatment effect

Two reviewers will use RevMan 5.3. independently to synthesize and statistically analyze the efficacy data. A risk ratio (RR) or odds ratio with 95% CIs will be employed for the dichotomous data, while a mean difference (MD) or standard mean difference (SMD) with 95% CIs will be utilized for the continuous data. SMD will be adopted if different assessment tools are used.

#### Dealing with missing data

If required data is elusive or missing, the reviewer will contact the corresponding author of the RCT reported by email. If data remains unobtainable, the reviewer will exclude the study. The potential impact of missing data will be assessed with a sensitivity analysis.

#### Assessment of heterogeneity

Heterogeneity will be assessed using Q-test and *I*^2^ statistic. The fixed-effects model will be adopted if the heterogeneity deems low (*I*^2^<50%), the random-effects model will be applied for moderate heterogeneity (50% < *I*^2^ <75%), and subgroup analysis or narrative analysis will be provided when the heterogeneity is high (*I*^2^>75%).

#### Data synthesis

In accordance with the Cochrane guideline, the fixed-effects model will be applied for the pooled data when the heterogeneity is low, the random-effect model will be utilized when heterogeneity is moderate, and a subgroup analysis or meta-regression will be conducted to investigate the potential sources if heterogeneity is considerably high. A *p*-value less than 0.05 is deemed as statistically significant. A narrative description will be provided when the meta-analysis is not feasible.

#### Subgroup analysis

If the necessary data are available, subgroup analyses will be conducted for the subtype of dysmenorrhea (primary/secondary), severity and duration of dysmenorrhea, footbaths alone versus control group, footbaths in combination with other treatments versus control group, and primary outcome measurements.

#### Sensitivity analysis

Sensitivity analysis will be carried out if feasible, and the robustness of the pooled results of the included RCTs will be assessed from such perspectives as the methodological quality, sample size, missing data, or high risk of bias.

#### Grading the quality of evidence

The Grading of Recommendations Assessment, Development and Evaluation (GRADE) will be applied to assess the strength of the evidence as high, moderate, low, or very low [38].

#### Ethics and dissemination plan

Ethical approval is unnecessary since this protocol is for systematic review and does not involve privacy data. Final reports of this study will be disseminated in peer-reviewed journals, the PROSPERO website, and relevant academic conferences. All data collected in the study, upon completion, will be made available with the publication of reports.

## Discussion

Dysmenorrhea is one of the most common gynecological complaints that impair women’s quality of life and effectiveness of day-to-day activities. Conventional treatment for dysmenorrhea such as NSAIDs may induce severe adverse events, and therefore an increasing number of patients worldwide are using TCM to relieve the ailment. Chinese herbal footbaths are a unique external therapy of TCM and have attracted increasing attention due to their effectiveness, fewer side effects, and favorable adherence [23, 39, 40]. It has been considered an ideal combination of heat therapy, reflex therapy, and Chinese herbal therapy. In recent years, several clinical trials have demonstrated the effectiveness and safety of Chinese herbal footbaths on the treatment of dysmenorrhea, indicating it may hold considerable promise for relieving dysmenorrhea. Herein, a critical evaluation and comprehensive synthesis of the available evidence will be performed in this systematic review to assess the efficacy and safety of Chinese herbal footbaths for the treatment of patients with dysmenorrhea. Hopefully, conclusions drawn from this study may benefit patients, physicians, and investigators concerned. The process of conducting this systematic review will include identification, study inclusion, data extraction, and data synthesis. If amendments were necessary, the date and statement of changes with corresponding reasons would be provided.

## Supporting information

S1 Checklist PRISMA 2009 checklist. (DOC)

## Abbreviations

CIs: confidence intervals
GRADE: Grading of Recommendations Assessment, Development and Evaluation
MD: mean difference
RCTs: randomized controlled trials
